# Association of Serum 25 (OH) Vitamin D With Chronic Kidney Disease Progression in Type 2 Diabetes

**DOI:** 10.3389/fendo.2022.929598

**Published:** 2022-06-30

**Authors:** Suyan Duan, Fang Lu, Buyun Wu, Chengning Zhang, Guangyan Nie, Lianqin Sun, Zhimin Huang, Honglei Guo, Bo Zhang, Changying Xing, Yanggang Yuan

**Affiliations:** Department of Nephrology, The First Affiliated Hospital of Nanjing Medical University, Nanjing Medical University, Nanjing, China

**Keywords:** 25-hydroxyvitamin D, type 2 diabetes mellitus, CKD progression, diabetic kidney disease, non-diabetic kidney disease

## Abstract

**Objectives:**

Growing evidence demonstrated that vitamin D levels had been linked to type 2 diabetes mellitus (T2DM) and chronic kidney disease (CKD) in light of various extraskeletal effects. Therefore, the present study aimed to evaluate the association of 25-hydroxyvitamin D [25(OH)D] level with the clinicopathological features and CKD progression in T2DM.

**Methods:**

A total of 182 patients with T2DM with CKD stages 1 through 4 (G1–G4) were retrospectively included. Identification of the serum 25(OH)D level associated with CKD progression was executed by Kaplan–Meier survival analysis and Cox proportional hazards models. We further performed sensitivity analyses with a time-weighted average (TWA) of the serum 25(OH)D level in 75 participants to reinforce the findings.

**Results:**

The median serum 25(OH)D level was 26 (IQR, 14; 39) nmol/L in the study participants. Median follow-up time was 42 months, during which 70 (38%) patients confronted CKD progression. Cumulative kidney outcomes were significantly higher in the lowest tertile of the serum 25(OH)D level in Kaplan–Meier analyses (*P <* 0.001). Consistently, the analyses of Cox proportional hazards regression models indicated a significantly greater risk for CKD progression in the lowest tertile of the serum 25(OH)D level compared with the highest tertile of the serum 25(OH)D level (*P =* 0.03). These relationships remained robust with further sensitivity analysis of data with TWA of the serum 25(OH)D level, showing an independent association between lower TWA of the serum 25(OH)D level and an unfavorable renal outcome in patients with T2DM with CKD.

**Conclusions:**

Our findings demonstrated that patients with T2DM with a decreased 25(OH)D level had deteriorated renal function. Both lower levels of baseline and TWA of serum 25(OH)D were associated with an increased risk of CKD progression in patients with T2DM, which suggested that the long-term maintenance of optimal vitamin D levels from early in life might be associated with reduced future risk of CKD development in T2DM.

## Introduction

The increasing prevalence of diabetes and its major microvascular complication, chronic kidney disease (CKD), has emerged globally as a substantial public health burden (1). The 10th edition of the International Diabetes Federation Atlas estimates that diabetes affected 537 million people in 2021 and is expected to reach 783 million by 2045, with the majority being type 2 diabetes mellitus (T2DM). Current data from the United States suggest that 37% of patients with diabetes were in coexistence with CKD stages 1 through 4 (G1–G4) and that 38% of end-stage kidney disease (ESKD) cases were on account of diabetes (2). In China, diabetes has been the primary cause of CKD since 2011, with an estimated 24.3 million diabetic patients living with CKD (3). Although significant progress has been made in the past three decades in the comprehensive treatment strategy, patients with T2DM remain at continuing high risk for progression of CKD, which is associated with increased cardiovascular complications, morbidity, and mortality (1, 2, 4). Individuals with T2DM plus superimposed CKD have been associated with approximately a three-fold increased risk of cardiovascular disease (CVD) and death than those with T2DM alone (5). Therefore, there is a compelling need to discover potential clinical indicators or prognostic factors to identify individuals at risk of CKD progression in T2DM who may benefit from early diagnosis and timely risk intervention in clinical practice.

As a pleiotropic steroid hormone, Vitamin D (VD) can exert various effects through binding to VD receptors (6). The primary function of VD is the regulation of calcium and phosphorus homeostasis to ensure adequate mineralization and bone growth (7). In addition, it plays a vital role in modulating cell proliferation, apoptosis, differentiation, inflammation response, immune function, and vascular and metabolic properties such as insulin secretion and insulin sensitivity (8–10). 25-*hydroxyvitamin D [25(OH)D] is synthesized by 25*-hydroxylase catalyzing VD, which is considered as the best indicator of VD status (11, 12). In recent years, lower serum 25(OH)D level has been implicated in the incidence of T2DM, which may rely on its association with impaired glucose and insulin metabolism (12, 13). More importantly, VD deficiency increases the risk of T2DM development and the incidence of its complication (14, 15). In particular, the prevalence of VD deficiency is very high in patients with CKD and the survival rates could be enhanced by active VD treatment (6, 16). A previous study reported that low 25(OH)D levels were associated with the development of ESKD (17). Nevertheless, it has not been fully elucidated about the relationship of the serum 25(OH)D level with kidney clinicopathologic features and renal outcomes in patients with T2DM. Furthermore, no literature has ever addressed the association between the time-weighted average (TWA) serum 25(OH)D level and CKD progression in patients with T2DM.

Hence, the current study set out to evaluate the significance of the serum 25(OH)D level for clinicopathological features and kidney progression, which was defined as a double increase in serum creatinine (D-Scr) from baseline values or occurrence of ESKD in T2DM with CKD. Further, to reinforce the point, the prospective association of TWA of the serum 25(OH)D level with the risk of CKD progression in T2DM was also investigated.

## Materials and Methods

### Subjects

**Figure 1:**
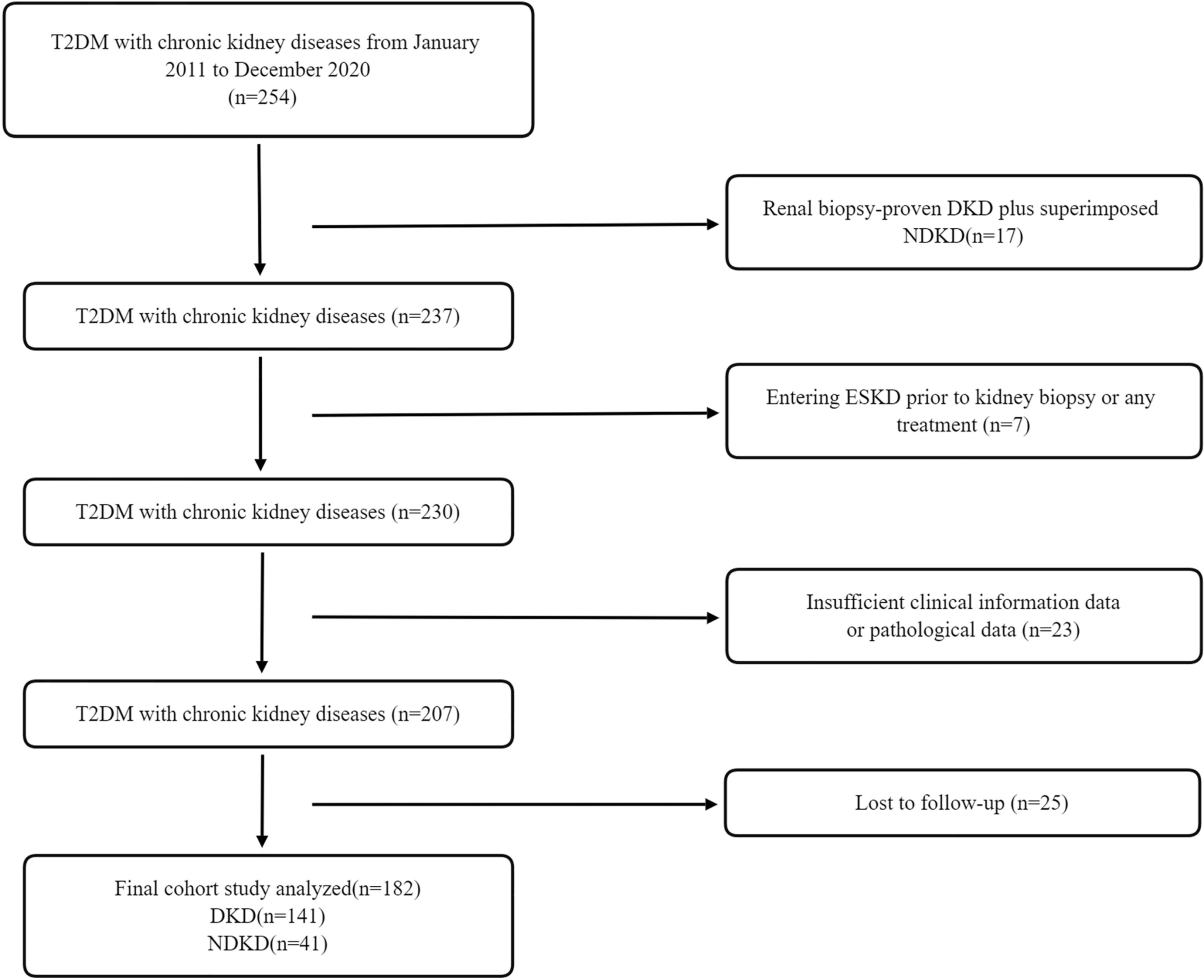
Flowchart of study participants. T2DM, type 2 diabetes mellitus; DKD, diabetic kidney disease; NDKD, non-diabetic kidney disease; CKD, chronic kidney disease; ESKD, end-stage kidney disease.

A total of 254 patients with diabetes mellitus (T2DM) with kidney diseases from January 2011 to December 2020 at the renal department of The First Affiliated Hospital of Nanjing Medical University were retrospectively reviewed. Finally, 182 patients were included and categorized into two groups: 141 patients with biopsy-proven diabetic kidney disease (DKD) and 41 patients with biopsy-proven non-DKD (NDKD) (**Figure 1**). T2DM was diagnosed according to the American Diabetes Association (18). The inclusion criteria were all patients diagnosed with T2DM complicated with CKD, defined as abnormalities of kidney structure or function, present for ≥3 months by the Kidney Disease:Improving Global Outcomes (KDIGO) Clinical Practice Guidelines. They had undergone renal biopsy pathological examination after excluding contraindications (19, 20). In addition, they should have intact information on the baseline serum 25(OH)D level. Exclusion criteria were as follows (1): advanced heart failure [the New York Heart Association (NYHA) functional classification III or IV]; (2) cirrhosis; (3) polycystic kidney disease; (4) other types of DM; (5) malignancies; (6) women with pregnancy; (7) acute inflammation or infections; (8) the estimated glomerular filtration rate (eGFR) ≤15 ml/min/1.73 m^2^; and (9) patients with new-onset diabetes after transplantation and those who underwent renal replacement therapy before the biopsy. This study was approved by the Ethics Committee of The First Affiliated Hospital of Nanjing Medical University.

### Clinical and Laboratory Parameters

The complete clinical and laboratory information of enrolled patients was collected at the time of renal biopsy, including age, gender, blood pressure, duration of diabetes, diabetic retinopathy, hypertension, body mass index (BMI), eGFR, blood urea nitrogen (BUN), serum creatinine (Scr), alkaline phosphatase (ALP), 25(OH)D, serum albumin, serum calcium and phosphorus, fasting blood glucose (FBG), glycosylated hemoglobin (HbA1c), triglyceride (TG), total cholesterol (TC), low-density lipoprotein cholesterol (LDL-C), high-density lipoprotein cholesterol (HDL-C), parathyroid hormone (PTH), 24-h urinary calcium and phosphorus excretion, serum immunoglobulin A (IgA), serum immunoglobulin G (IgG), complement C3, complement C4, serum and urinary neutrophil gelatinase-associated lipocalin (NAGL), urinary N-acetyl-β-D glucosaminidase (uNAG), and retinol-binding protein (RBP).

Serum 25(OH)D measurements in the follow-up period were averaged into TWA serum 25(OH)D level for each patient. The TWA value was derived as an aggregate area under the curve divided by the cumulative time exposure for each patient. The area under the curve was measured as an integrated expression over time using a positive incremental method, without imputation for missing time points. The calculated formula is as follows: TWA serum 25(OH)D = {[(X_1_ + X_2_) (T_2_ − T_1_) + (X_2_ + X_3_) (T_3_ − T_2_) + … +(X_n−1_ + X_n_) (T_n_ − T_n−1_)]/[2×(T_n_ − T_1_)]}, where T_n_ is nth time point and X_n_ is the serum 25(OH)D level at T_n_ (21).

The current medications of participants were also recorded, including blood pressure–lowering therapy [renin-angiotensin-aldosterone system inhibitor (RAASi), β-blocker, diuretic and calcium-channel blocker (CCB), statins, insulin, and oral hypoglycemic agents]. The serum 25(OH)D level was determined by electrochemiluminescence immunoassay (Roche Diagnostic GmBH, Germany). According to the Endocrine Society clinical practice (ESC) guidelines (22) and the UK Scientific Advisory Committee on Nutrition (SACN) (23), VD status was defined as follows: severely deficient for 25(OH)D <25 nmol/L, deficient for 25 nmol/L≤ 25(OH)D <50 nmol/L, insufficient for 50 nmol/L ≤25(OH)D <75 nmol/L, and sufficient for 25(OH)D ≥75 nmol/L. The Institute of Medicine (IOM) established serum VD values ≥50 nmol/L as sufficient, values between 30 and 50 nmol/L as insufficiency, and values <30 nmol/L as deficiency (24). eGFR was estimated using Chronic Kidney Disease Epidemiology Collaboration (CKD-EPI) equation (25). In addition, CKD stage was evaluated according to the K/DOQI guidelines.

### Kidney Histopathology

Routine examination of every renal biopsy specimen was performed by light microscopy, electron microscopy, and immunofluorescence. Glomerular, tubulointerstitial, and vascular lesions were scored according to the DKD pathologic classification (26). The glomerular classifications were as follows: class I, glomerular basement membrane (GBM) thickening; class IIa, mild mesangial expansion; class IIb, severe mesangial expansion; class III, nodular sclerosis; and class IV, global glomerulosclerosis in >50% of glomeruli. Semi-quantitative scores for interstitial fibrosis and tubular atrophy (IFTA) were obtained according to the affected proportion of the tubulointerstitial compartment (0, none; 1, <25%; 2, 25%–50%; and 3, >50%), and the scale of interstitial inflammation (0, absent; 1, infiltration only in areas related to IFTA; and 2, infiltration in areas without IFTA). Scores for vascular lesions were based on large-vessel arteriosclerosis and arteriolar hyalinosis. Semi-quantitative rank for the intensity of IgG, IgA, IgM, complement 1q (C1q), C3, and C4 staining in each renal tissue section by direct immunofluorescence was classified into four categories (0, negative; 1, weak staining; 2, moderate staining; and 3, strong staining). Any scoring differences between two pathologists were repeatedly reviewed until a consensus was obtained.

### Kidney Outcomes

The primary outcome was the composite kidney outcome, defined as a double increase in serum creatinine (D-Scr) from baseline values or the occurrence of ESKD. ESKD was defined as the initiation of maintenance dialysis or kidney transplantation. Patients who did not reach the endpoint were recorded using the information of their last follow-up visit. Survival time was calculated from the enrollment to the occurrence of the event or the last follow-up. Patient visits usually occurred at intervals of 3–6 months except for those with CKD stage 3 and stage 4 who were under close observation and followed at 1–3 months.

### Statistical Analysis

We divided the study population into tertiles according to baseline serum 25(OH)D level. Data were presented as mean ± SD, median and interquartile range, or percentage. As appropriate, comparisons between groups were performed using one-way analysis of variance (ANOVA), Kruskal–Wallis test, or χ2 test. Pearson’s or Spearman correlations were calculated to characterize the associations between baseline characteristics and serum 25(OH)D level. Kaplan–Meier analysis and the log-rank test were used to assess renal survival differences among groups. Hazard ratios for the serum 25(OH)D level with CKD progression were estimated using Cox proportional hazards regression models with follow-up time. The assumption of proportionality was tested using Schoenfeld residuals and interaction terms with time for each exposure variable and covariate. In addition, multiple covariables were adjusted. In addition, we further performed sensitivity analyses with TWA of the serum 25(OH)D level in 75 participants to reinforce the findings. Kaplan–Meier analysis and Cox proportional hazards regression models were performed to evaluate the effect of TWA of the serum 25(OH)D level on renal outcomes. A *p*-value of <0.05 was considered statistically significant. All statistics were done in IBM SPSS v.24.0 and R v.4.0.2.

## Results

### Baseline Clinical and Pathologic Characteristics According to Tertiles of the Serum 25(OH)D Level

In total, 182 patients with T2DM with DKD (141, 78%) and NDKD (41, 22%) were enrolled in this study (**Figure 1**). During a median follow-up time of 42 months (IQR, 24; 62 months), 70 incident kidney outcomes were identified. The clinical characteristics of the cases divided into two groups according to the later development of renal endpoints were summarized in **Table 1**. The mean age was 52 ± 11 years old, and most were male (75%). CKD stages and pathological types were significantly distributed between the two groups (no incidence versus the incidence of renal endpoints, *P <* 0.001). Compared with patients without renal outcomes, significantly higher levels of systolic blood pressure, urinary protein, serum creatinine (Scr), blood urea nitrogen (BUN), total cholesterol, LDL-C, HDL-C, C3, C4, PTH, serum NAGL, urinary NAGL, and 24-h uNAG were observed in patients with D-Scr or development of ESKD, along with lower levels of eGFR, serum albumin, hemoglobin, 25(OH)D, and serum calcium. Moreover, RAASi and oral hypoglycemic agents were significantly more prevalent among patients without D-Scr or ESKD. Insulin, β-blocker, and CCB were used more in patients with D-Scr or ESKD.

**Table 1:** Baseline characteristics and laboratory data for all enrolled patients.

Parameter	Total (n = 182)	D-Scr or ESKD (n = 70)	No D-Scr and no ESKD (n = 112)	*P*-value
Age (years)	52 ± 11	49 ± 11	54 ± 11	**0.005**
Gender (male/female)	137/45	55/15	82/30	0.52
Duration of diabetes (years)	8.0 (3.0, 14.0)	9.0 (3.3, 14.5)	8.0 (2.0, 14.5)	0.73
Composite renal outcome (%)	70 (38)	70 (100)	0 (0)	**<0.001**
Double of serum creatinine (%)	7 (3.8)	7 (10)	0 (0)	**<0.001**
ESKD (%)	63 (35)	63 (90)	0 (0)	**<0.001**
**Comorbid disease**				
CKD stage (1/2/3a/3b/4)	43/60/23/27/29	8/19/10/12/21	35/41/13/15/8	**<0.001**
DKD/NDKD (cases)	141/41	68/2	73/39	**<0.001**
Diabetic retinopathy (%)	45 (25)	21 (30)	24 (21)	0.26
Diabetic neuropathy (%)	13 (7.1)	7 (10.0)	6 (5.0)	0.38
Cardiovascular diseases (%)	17 (9.3)	2 (2.8)	15 (13.4)	**0.03**
Hypertension (%)	136 (75)	51 (73)	85 (76)	0.78
**Clinical parameter**				
Body mass index (kg/m2)	25 (23, 27)	25 (23, 27)	25 (23, 27)	0.42
SBP (mmHg)	140 (130, 157)	144 (133, 163)	139 (126, 154)	**0.050**
DBP (mmHg)	85 (74, 94)	85 (78, 95)	83 (74, 92)	0.20
**Laboratory parameter**				
Urinary protein (g/d)	2.9 (1.3, 7.0)	5.5 (2.9, 9.0)	2.0 (0.8, 5.8)	**<0.001**
eGFR (ml/min/1.73 m²)	65 (38, 88)	51 (28, 73)	72 (47, 97)	**<0.001**
BUN (mmol/L)	8.9 (6.5, 11.6)	6.6 (5.0, 9.0)	9.9 (7.5, 11.6)	**<0.001**
Scr (μmol/L)	108 (82, 171)	145 (100, 213)	97 (71, 133)	**<0.001**
Uric acid (mmol/L)	369 (316, 425)	388 (335, 442)	357 (315, 418)	0.08
Serum albumin (g/L)	32 ± 7.9	30 ± 7.2	33 ± 8.1	**0.01**
ALP (U/L)	83 (61, 105)	88 (70, 105)	78 (59, 102)	0.28
Fasting blood glucose (mmol/L)	6.4 (4.8, 8.3)	6.9 (5.1, 8.3)	6.3 (4.6, 8.2)	0.32
Glycosylated hemoglobin (%)	7.1 (6.3, 8.1)	6.7 (6.2, 7.7)	7.2 (6.5, 8.6)	**0.04**
Triglyceride (mmol/L)	1.5 (1.1, 2.3)	1.7 (1.1, 2.3)	1.5 (1.1, 2.5)	0.70
Total cholesterol (mmol/L)	5.0 (4.0, 6.0)	5.3 (4.4, 6.4)	4.8 (3.8, 5.9)	**0.02**
LDL-C (mmol/L)	3.2 (2.6, 3.9)	3.5 (2.8, 4.3)	3.1 (2.5, 3.7)	**0.008**
HDL-C (mmol/L)	1.04 (0.87, 1.31)	1.10 (0.93, 1.43)	1.00 (0.85, 1.24)	**0.04**
Hemoglobin (g/L)	115 ± 23	106 ± 18	120 ± 24	**< 0.001**
IgA (g/L)	2.4 (1.8, 3.0)	2.4 (2.0, 3.0)	2.4 (1.7, 3.0)	0.44
IgG (g/L)	9.6 (7.6, 12)	9.5 (7.1, 11)	9.9 (7.7, 12)	0.28
C3 (g/L)	1.05 (0.95, 1.18)	1.06 (0.98, 1.21)	1.03 (0.90, 1.17)	**0.04**
C4 (g/L)	0.30 ± 0.10	0.31 ± 0.08	0.27 ± 0.09	**0.02**
PTH (pg/ml)	44 (27, 70)	49 (32, 82)	37 (24, 53)	**0.01**
25(OH)D (nmol/L)	26 (14, 39)	16 (10, 30)	32 (20, 46)	**<0.001**
Serum calcium (mmol/L)	2.13 (2.00, 2.23)	2.07 (1.96, 2.17)	2.16 (2.04, 2.27)	**0.004**
Serum phosphorus (mmol/L)	1.23 (1.12, 1.38)	1.26 (1.15, 1.42)	1.22 (1.12, 1.37)	0.37
24-h urinary calcium (mmol/d)	1.60 (0.88, 3.18)	1.47 (0.72, 2.66)	1.62 (0.94, 3.64)	0.24
24-h urinary phosphorus (mmol/d)	16.7 (12.3, 23.0)	16.5 (13.0, 19.6)	16.7 (11.6, 23.6)	0.86
Serum NAGL (ng/mL)	218 (141, 304)	243 (183, 414)	180 (126, 266)	**0.003**
Urinary NAGL (ng/mL)	53 (25, 92)	78 (25, 194)	25 (25, 71)	**<0.001**
24-h uNAG (U/L)	15 (10, 24)	20 (14, 28)	14 (9.3, 23)	**0.002**
RBP (mg/L)	60 (43, 73)	66 (55, 84)	56 (40, 69)	**0.002**
**Medications**	
RAAS inhibitor (%)	147 (81)	46 (66)	101 (90)	**< 0.001**
Oral hypoglycemic agents (%)	90 (50)	24 (34)	66 (59)	**0.002**
Insulin (%)	129 (71)	59 (84)	70 (63)	**0.003**
β-blocker (%)	52 (29)	29 (41)	23 (21)	**0.004**
Diuretic (%)	21 (12)	8 (11)	13 (12)	1.00
CCB (%)	118 (65)	54 (77)	64 (57)	**0.01**
Statins (%)	78 (43)	27 (39)	51 (46)	0.44
Calcium supplements (%)	30 (16)	15 (21)	15 (13)	0.22

DKD, diabetic kidney disease; NDKD, non-diabetic kidney disease; D-Scr, doubling of serum creatinine level; ESKD, end-stage kidney disease; BMI, body mass index; SBP, systolic blood pressure; DBP, diastolic blood pressure; eGFR, estimated glomerular filtration rate; BUN, blood urea nitrogen; Scr, serum creatinine; HDL-C, high-density lipoprotein cholesterol; LDL-C, low-density lipoprotein cholesterol; TG, triglyceride; TC, total cholesterol; IgA, immunoglobulin A; IgG, immunoglobulin G; C3, complement 3; C4, complement 4; 24-h UV, 24-h urinary volume; PTH, parathyroid hormone; 25(OH)D, 25-hydroxy vitamin D; RAAS, renin-angiotensin-aldosterone system; CCB, calcium-channel blocker.

Data were presented as the mean ± standard, the median with interquartile range, or counts and percentages. A two-tailed P < 0.05 was considered statistically significant and presented in bold.

The clinical characteristics of the study population according to tertiles of the serum 25(OH)D levels are presented in **Table 2**. The median serum 25(OH)D level was 26 (IQR, 14; 39) nmol/L in the study participants. During follow-up, the incidence of composite kidney outcomes in the lowest tertile was the highest among groups (*P <* 0.001), with 35 patients (58%) progressing to ESKD and four patients (6.7%) progressing to D-Scr from the time of renal biopsy. Consistently, kidney function was better when the serum 25(OH)D level was higher, wherein eGFR was significantly increased in the highest tierce, which had the lowest levels of Scr, BUN, and uric acid (all *P <* 0.05). In addition, blood pressure (including systolic blood pressure, diastolic blood pressure, and mean arterial pressure), CKD stages, 24-h urinary protein, levels of TC, LDL-C, and urinary NAGL reduced, whereas levels of serum albumin, hemoglobin, serum calcium, and 24-h urinary calcium rose across the increasing tertile of serum 25(OH)D (all *P <* 0.05). Moreover, significant differences in the current use of RAASi, oral hypoglycemic agents, diuretic, and statins were observed among the tertiles at baseline (all *P <* 0.05).

**Table 2:** Clinical characteristics and laboratory findings of all enrolled patients according to tertiles of vitamin D level.

Parameter	Tertiles of the serum 25(OH)D level (nmol/L)			*P*-value
	T1 (≤17) (n = 60)	T2 (17-35) (n = 62)	T3 (>35) (n = 60)	
Age (years)	51 ± 12	52 ± 12	54 ± 9.7	0.32
Gender (male/female)	45/15	45/17	47/13	0.79
**Comorbid disease**				
CKD stage (1/2/3a/3b/4)	7/17/11/12/13	15/20/7/10/10	21/23/5/5/6	**0.001**
Duration of diabetes (years)	8.5 (3.0, 14)	10 (4.3, 12)	7.0 (3.8, 15)	0.91
Diabetic retinopathy (%)	19 (32)	15 (24)	11 (18)	0.25
Diabetic neuropathy (%)	7 (12)	3 (4.8)	3 (5.0)	0.29
Hypertension (%)	39 (65)	49 (79)	48 (80)	0.11
Cardiovascular diseases (%)	1 (1.7)	8 (13)	8 (13)	**0.04**
**Clinical parameter**				
BMI (kg/m^2^)	25 (23, 27)	24 (23, 27)	26 (24, 28)	0.14
SBP (mmHg)	146 (136, 167)	140 (127, 155)	135 (127, 145)	**0.005**
DBP (mmHg)	87 (79, 99)	83 (72, 90)	81 (73, 93)	**0.01**
MAP (mmHg)	109 ± 16	102 ± 14	101 ± 12	**0.002**
**Laboratory parameter**				
Urinary protein excretion (g/d)	6.9 (4.7, 11)	2.9 (1.7, 5.6)	1.1 (0.31, 2.7)	**<0.001**
eGFR (ml/min/1.73 m²)	53 (32, 72)	66 (38, 87)	78 (55, 99)	**0.001**
BUN (mmol/L)	9.3 (7.4, 12)	9.5 (6.5, 13)	7.7 (5.7, 11)	**0.04**
Scr (μmol/L)	129 (101, 198)	108 (82, 171)	93 (71, 124)	**0.001**
Uric acid (mmol/L)	350 (314, 415)	397 (347, 472)	351 (312, 417)	**0.02**
Serum albumin (g/L)	26 ± 6.6	33 ± 6.1	38 ± 5.9	**<0.001**
ALP (U/L)	88 (67, 106)	74 (61, 92)	87 (62, 100)	0.44
Serum 25(OH)D (nmol/L)	10 (7.8, 14)	26 (22, 30)	47 (39, 60)	**<0.001**
Serum calcium (mmol/L)	1.97 (1.89, 2.11)	2.15 (2.04, 2.21)	2.24 (2.15, 2.30)	**<0.001**
Serum phosphorus (mmol/L)	1.22 (1.06, 1.38)	1.27 (1.15, 1.46)	1.19 (1.12, 1.34)	0.25
24-h urinary calcium (mmol/d)	0.99 (0.66, 2.24)	1.45 (0.86, 2.28)	2.41 (1.40, 4.36)	**0.002**
24-h urinary phosphorus (mmol/d)	12.3 (9.0, 16.9)	17.9 (14.2, 23.9)	17.1 (13.2, 24.0)	**0.001**
FBG (mmol/L)	6.6 (5.1, 10)	6.2 (4.6, 8.2)	6.3 (4.7, 7.9)	0.44
HbA1c (%)	6.8 (6.1, 8.1)	7.1 (6.5, 8.4)	7.1 (6.3, 8.0)	0.49
TG (mmol/L)	1.7 (1.2, 2.6)	1.6 (1.3, 2.4)	1.4 (1.0, 2.1)	0.12
TC (mmol/L)	5.7 (5.0, 7.1)	5.2 (4.2, 6.0)	4.1 (3.5, 4.9)	**<0.001**
LDL-C (mmol/L)	3.8 (3.1, 4.9)	3.3 (2.7, 4.0)	2.6 (2.0, 3.2)	**<0.001**
HDL-C (mmol/L)	1.11 (0.97, 1.48)	0.99 (0.84, 1.24)	1.01 (0.86, 1.22)	**0.02**
Hemoglobin (g/L)	105 ± 20	115 ± 23	123 ± 21	**<0.001**
PTH (pg/mL)	57 (34, 100)	42 (28, 62)	36 (23, 46)	**0.007**
Serum IgA (g/L)	2.5 (1.9, 3.1)	2.2 (1.7, 2.7)	2.5 (1.8, 3.1)	0.16
Serum IgG (g/L)	7.5 (6.1, 9.9)	10 (7.8, 13)	11 (9.1, 14)	**<0.001**
Serum C3 (g/L)	1.08 (0.98, 1.20)	1.03 (0.86, 1.13)	1.04 (0.94, 1.19)	0.10
Serum C4 (g/L)	0.31 ± 0.09	0.26 ± 0.09	0.28 ± 0.08	**0.01**
Serum NAGL (ng/mL)	228 (115, 390)	222 (171, 362)	159 (135, 250)	0.21
Urinary NAGL (ng/mL)	95 (57, 190)	53 (25, 77)	25 (25, 38)	**<0.001**
24-h uNAG (U/L)	22 (18, 36)	15 (10, 25)	12 (8.2, 17)	**<0.001**
RBP (mg/L)	58 (44, 71)	62 (48, 76)	58 (41, 72)	0.57
**Medications**				
RAAS inhibitor (%)	42 (70)	53 (86)	52 (87)	**0.04**
Oral hypoglycemic agents (%)	17 (28)	34 (55)	39 (65)	**<0.001**
Insulin (%)	47 (78)	45 (73)	37 (62)	0.12
β-blocker (%)	22 (37)	17 (27)	13 (22)	0.19
Diuretic (%)	13 (22)	5 (8.1)	3 (5.0)	**0.01**
CCB (%)	44 (73)	38 (61)	36 (60)	0.24
Statins (%)	30 (50)	30 (48)	18 (30)	**0.048**
Calcium supplements (%)	13 (22)	12 (19)	5 (8.3)	0.11
**Progression**				
Composite renal outcome (%)	39 (65)	20 (32)	11 (18)	**<0.001**
D-Scr (%)	4 (6.7)	1 (1.6)	2 (3.3)	0.31
ESKD (%)	35 (58)	19 (31)	9 (15)	**<0.001**

CKD, chronic kidney disease; BMI, body mass index; SBP, systolic blood pressure; DBP, diastolic blood pressure; MAP = (systolic blood pressure+2×diastolic blood pressure)/3; eGFR, estimated glomerular filtration rate; BUN, blood urea nitrogen; Scr, serum creatinine; ALP, alkaline phosphatase; 25(OH)D, 25-hydroxyvitamin D; FBG, fasting blood glucose; HbA1c, glycosylated hemoglobin; TG, triglyceride; TC, total cholesterol; LDL-C, low-density lipoprotein cholesterol; HDL-C, high-density lipoprotein cholesterol; PTH, parathyroid hormone; IgA, immunoglobulin A; IgG, immunoglobulin G; C3, complement 3; C4, complement 4; NAGL, neutrophil gelatinase-associated lipocalin; uNAG, urinary N-acetyl-β-D glucosaminidase; RBP, retinol-binding protein; RAAS, renin-angiotensin-aldosterone system; CCB, calcium-channel blocker. D-Scr, doubling of serum creatinine level; ESKD, end-stage kidney disease.

Data were presented as the mean ± standard, the median with interquartile range, or counts and percentages. A two-tailed P < 0.05 was considered statistically significant and presented in bold.

**Figure 2:**
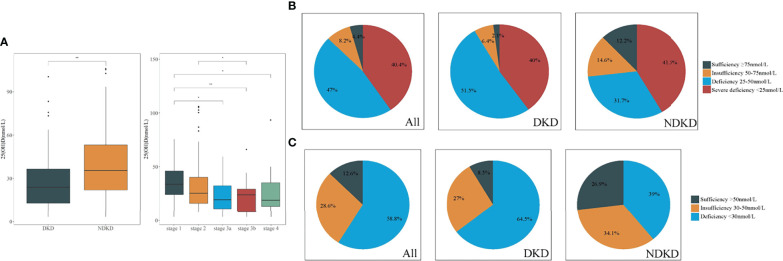
Comparison of vitamin D levels in patients with DKD and NDKD. **(A)** Significant difference was obtained in vitamin D levels between DKD and NDKD, in different stages of CKD, respectively. **(B, C)**. Vitamin D status in all patients with DKD and NDKD, respectively. **(B)** Four categories according to the Endocrine Society clinical practice (ESC) guidelines and the UK Scientific Advisory Committee on Nutrition (SACN). **(C)** Three categories according to the Institute of Medicine (IOM). *P < 0.05 between all groups. **P < 0.01 between groups. DKD, diabetic kidney disease; NDKD, non-diabetic kidney disease; 25(OH)D, 25-hydroxyvitamin D.

In terms of pathological types, patients with DKD had significantly lower serum 25(OH)D levels than those with NDKD (*P <* 0.01, Wilcoxon test, **Figure 2A**). Moreover, there was a significant decrease in 25(OH)D at stage G1 relative to later CKD stages (G1 vs. G3a: *P <* 0.05; G1 vs. G3b: *P <* 0.01; G1 vs. G4: *P <* 0.05). Moreover, only eight (4.4%) patients and 15 (8.2%) patients showed sufficient (≥75 nmol/L) and insufficient 25(OH)D levels (50–75 nmol/L) according to the ESC guideline, with 12.6% exhibiting 25(OH)D sufficiency (≥50 nmol/L) according to the IOM guideline. The prevalence of VD deficiency (25–50 nmol/L) in patients with DKD was higher than that in patients with NDKD (51.5% vs. 31.7%), whereas VD severe deficiency (<25 nmol/L) ratios were similar between two groups (40% vs. 41.5%) in accordance with the ESC and SACN guidelines (**Figure 2B**). In addition, with the standard of IOM guideline, 64.5% of patients with DKD were at risk of deficiency relative to bone health (<30 nmol/L), higher than patients with NDKD (39%) (**Figure 2C**). **Table 3** displays the baseline pathological features of the recruited study population, both overall and stratified by the serum 25(OH)D level. DKD was common in the lowest tertile, whereas NDKD in the highest. In addition, there were significant differences in the glomerular class of DKD, pathological classification of NDKD, and vascular lesion score among groups (all *P <* 0.05). Moreover, patients in the lowest tertile of serum 25(OH)D tended to have a greater proportion of glomerular IgM, C3, and C4 deposition, especially in DKD cases (all *P <* 0.05). On the other hand, those with the highest tertile of serum 25(OH)D tended to have stronger staining of glomerular IgA deposition, especially in NDKD cases (all *P <* 0.05).

**Table 3:** Pathological features of all enrolled patients according to tertiles of vitamin D level.

Pathological feature	Total (n = 182)	Tertiles of the serum 25(OH)D level (nmol/L)			*P*-value
		T1 (≤17) (n = 60)	T2 (17-35) (n = 62)	T3 (>35) (n = 60)	
**DKD/NDKD (cases)**	141/41	53/7	49/13	39/21	**0.009**
**Glomerular class of DKD (I/IIa/IIb/III/IV)**	0/12/33/73/23	0/1/10/35/7	0/4/9/25/11	0/7/14/13/5	**0.01**
**Pathological** **classification of NDKD**					
IgAN/MCD/FSGS/MN/LN/CGN (cases)	18/1/9/9/1/3	1/0/2/4/0/0	3/0/3/4/1/2	14/1/4/1/0/1	**0.004**
**IFTA Score (0/1/2/3)**	12/65/37/66	2/18/12/26	6/21/14/21	4/26/12/19	0.18
DKD subtype	6/40/34/61	2/14/12/25	2/13/14/20	2/13/8/16	0.74
NDKD subtype	6/25/3/5	0/4/0/1	4/8/0/1	2/13/3/3	0.14
**Interstitial inflammation (0/1/2/3)**	23/101/55/1	10/25/22/1	5/43/14/0	8/33/19/0	0.63
DKD subtype	17/79/44/1	8/24/20/1	4/33/12/0	5/22/12/0	0.65
NDKD subtype	6/22/11/0	2/1/2/0	1/10/2/0	3/11/7/0	0.80
**Vascular lesion Score (0/1/2)**	38/58/84	8/21/29	11/16/35	19/21/20	**0.02**
DKD subtype	13/44/84	5/19/29	2/12/35	6/13/20	0.08
NDKD subtype	25/14/0	3/2/0	9/4/0	13/8/0	0.89
**Global sclerosis, %**	25 (7.3, 42)	26 (13, 39)	22 (6.9, 50)	28 (11, 46)	0.74
DKD subtype	25 (9.6, 45)	25 (12, 39)	20 (6.9, 46)	30 (12, 46)	0.62
NDKD subtype	21 (0.0, 33)	32 (31, 43)	15 (6.7, 33)	20 (0.0, 33)	0.44
**Glomerular IgG deposition (0/1/2/3)**	119/45/9/6	34/17/6/1	40/16/2/3	45/12/1/2	0.16
DKD subtype	93/40/7/1	32/16/4/1	33/14/2/0	28/10/1/0	0.41
NDKD subtype	26/5/2/5	2/1/2/0	7/2/0/2	17/2/0/2	0.18
**Glomerular IgM deposition (0/1/2/3)**	39/31/42/67	6/8/13/31	17/12/9/23	16/11/20/13	**0.002**
DKD subtype	29/22/33/57	5/5/13/30	14/11/6/18	10/6/14/9	**0.002**
NDKD subtype	10/9/9/10	1/3/0/1	3/1/3/5	6/5/6/4	0.47
**Glomerular IgA deposition (0/1/2/3)**	103/32/19/25	34/14/6/4	41/5/10/5	28/13/3/16	**0.046**
DKD subtype	88/31/14/8	31/13/6/3	35/5/6/3	22/13/2/2	0.51
NDKD subtype	15/1/5/17	3/1/0/1	6/0/4/2	6/0/1/14	**0.046**
**Glomerular C3 deposition (0/1/2/3)**	93/17/16/53	19/8/6/25	38/5/5/13	36/4/5/15	**0.002**
DKD subtype	73/13/14/41	15/7/6/25	34/3/5/7	24/3/3/9	**<0.001**
NDKD subtype	20/4/2/12	4/1/0/0	4/2/0/6	12/1/2/6	0.21
**Glomerular C4 deposition (0/1/2/3)**	136/18/13/12	39/6/6/7	45/10/3/3	52/2/4/2	**0.04**
DKD subtype	103/16/11/11	34/6/6/7	35/9/3/2	34/1/2/2	**0.049**
NDKD subtype	33/2/2/1/	5/0/0/0	10/1/0/1	18/1/2/0	0.64
**Glomerular C1q deposition (0/1/2/3)**	112/27/21/19	31/9/9/9	38/13/3/7	43/5/9/3	0.11
DKD subtype	87/19/18/17	27/8/9/9	34/7/2/6	26/4/7/2	0.10
NDKD subtype	25/8/3/2	4/1/0/0	4/6/1/1	17/1/2/1	**0.04**

DKD, diabetic kidney disease; NDKD, non-diabetic kidney disease; IgAN, IgA nephropathy; MCD, minimal change disease; FSGS, focal segmental glomerulosclerosis; MN, membranous nephropathy; LN, lupus nephritis; CGN, crescentic glomerulonephritis; IFTA, interstitial inflammation.

A two-tailed P < 0.05 was considered statistically significant and presented in bold.

### Correlation Between the Serum 25(OH)D Level and Clinicopathological Characteristics

On further analyses by Spearman test (**Table 4**), the serum 25(OH)D level was negatively correlated with proteinuria (r = −0.62, *P <* 0.001), Scr (r = −0.26, *P <* 0.001), BUN (r = −0.17, *P =* 0.02), TC (r = −0.48, *P <* 0.001), LDL-C (r = −0.46, *P <* 0.001), HDL-C (r = −0.19, *P =* 0.01), PTH (r = −0.20, *P =* 0.02), serum phosphorus (r = −0.24, *P =* 0.008), serum C4 level (r = −0.17, *P =* 0.03), 24-h uNAG (r = −0.49, *P <* 0.001), CKD stage (r = −0.26, *P <* 0.001), and vascular lesion score (r = −0.16, *P =* 0.04), whereas it was positively correlated with eGFR (r = 0.26, *P <* 0.001), serum albumin (r = 0.66, *P <* 0.001), serum calcium (r = 0.28, *P =* 0.002), serum IgG (r = 0.46, *P <* 0.001), 24-h urinary calcium (r = 0.28, *P =* 0.002), and 24-h urinary phosphorus (r = 0.24, *P =* 0.008).

**Table 4:** Correlations between serum 25(OH)D and clinicopathological parameters.

Parameter	25(OH)D	
	r	*P*-value
**Clinical parameter**		
Urinary protein excretion (g/d)	−0.62	**<0.001**
eGFR (ml/min/1.73 m²)	0.26	**<0.001**
BUN (mmol/L)	−0.17	**0.02**
Scr (μmol/L)	−0.26	**<0.001**
Serum albumin (g/L)	0.66	**<0.001**
FBG (mmol/L)	−0.13	0.08
HbA1c (%)	−0.03	0.69
TG (mmol/L)	−0.15	0.05
TC(mmol/L)	−0.48	**<0.001**
LDL-C (mmol/L)	−0.46	**<0.001**
HDL-C (mmol/L)	−0.19	**0.01**
PTH(pg/mL)	−0.20	**0.02**
Serum calcium (mmol/L)	0.28	**0.002**
Serum phosphorus (mmol/L)	−0.24	**0.008**
Serum IgA (g/L)	−0.01	0.85
Serum IgG (g/L)	0.46	**<0.001**
Serum C3 (g/L)	−0.09	0.25
Serum C4 (g/L)	−0.17	**0.03**
Serum NAGL (ng/mL)	−0.08	0.40
Urinary NAGL (ng/mL)	−0.48	**<0.001**
24-h uNAG (U/L)	−0.49	**<0.001**
24-h urinary calcium (mmol/d)	0.28	**0.002**
24-h urinary phosphorus (mmol/d)	0.24	**0.008**
RBP (mg/L)	−0.003	0.97
CKD stage (1/2/3a/3b/4)	−0.26	**<0.001**
**Pathological feature**		
IFTA Score (0/1/2/3)	−0.12	0.13
Interstitial inflammation (0/1/2/3)	−0.03	0.65
Vascular lesion Score (0/1/2)	−0.16	**0.04**
Global sclerosis, %	0.04	0.63

DKD, diabetic kidney disease; eGFR, estimated glomerular filtration rate; BUN, blood urea nitrogen; Scr, serum creatinine; FBG, fasting blood glucose; HbA1c, glycosylated hemoglobin; TG, triglyceride; TC, total cholesterol; LDL-C, low-density lipoprotein cholesterol; HDL-C, high-density lipoprotein cholesterol; IgA, immunoglobulin A; IgG, immunoglobulin G; C3, complement 3; C4, complement 4; NAGL, neutrophil gelatinase-associated lipocalin; uNAG, urinary N-acetyl-β-D glucosaminidase; RBP, retinol-binding protein; IFTA, interstitial inflammation.

A two-tailed P < 0.05 was considered statistically significant and presented in bold.

### Effect of the Serum 25(OH)D Level on Kidney Outcomes

**Figure 3:**
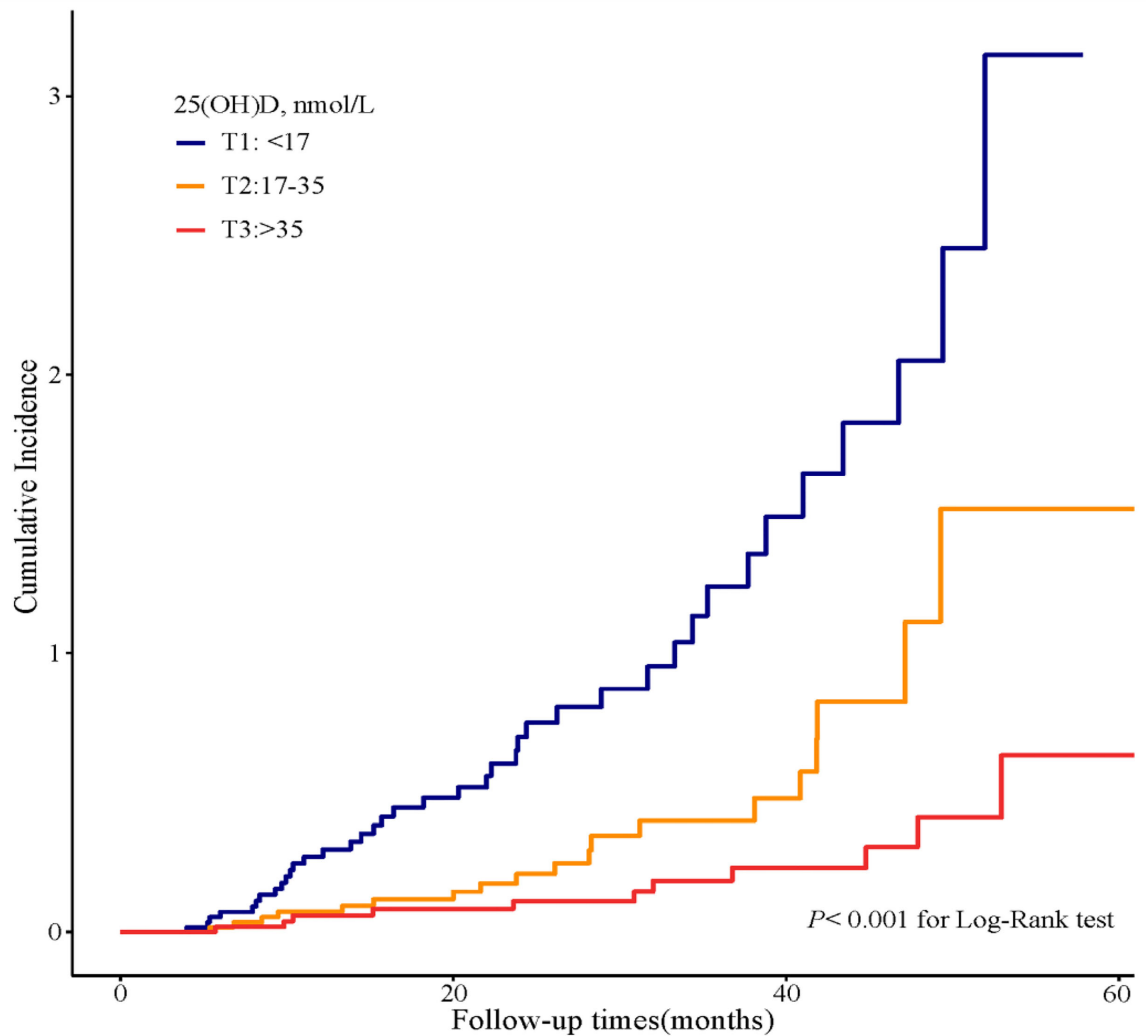
Cumulative incidence of CKD progression in strata of tertiles of the serum 25(OH)D levels. Kaplan–Meier curves comparing different strata of tertiles of the serum 25(OH)D levels in enrolled patients.

**Figure 4:**
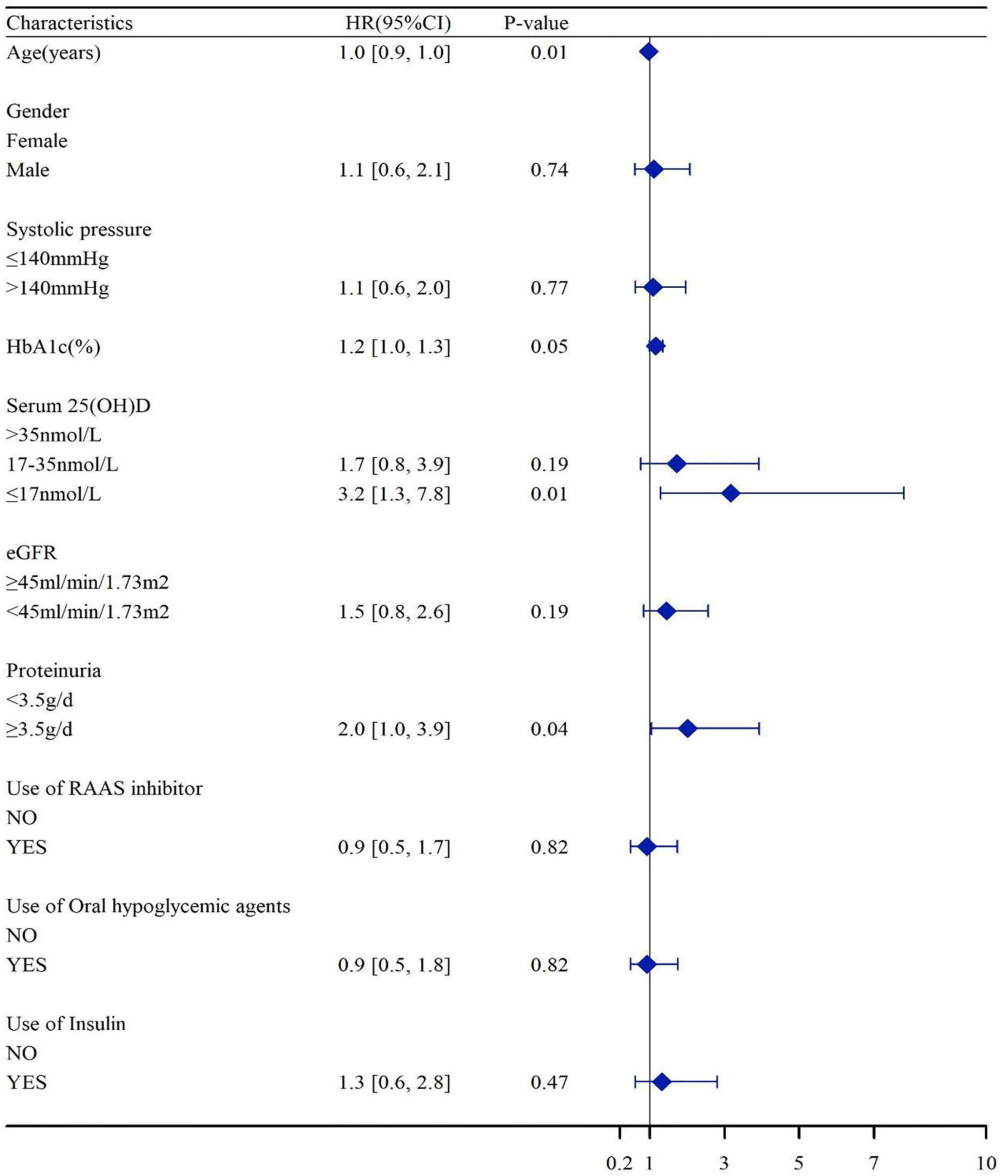
Association of the serum 25(OH)D levels with HR of CKD progressions. Hazard ratios were adjusted for baseline age, gender, HbA1c, 24-h urinary protein, systolic blood pressure, use of RAASi, oral hypoglycemic agents, and insulin. eGFR, estimated glomerular filtration rate; HbA1c, glycosylated hemoglobin; RAASi, renin-angiotensin-aldosterone system inhibitor.

In the Kaplan–Meier survival analysis (**Figure 3**), the cumulative incidence of kidney outcomes decreased across increasing tertiles of serum 25(OH)D (*P <* 0.001), which suggested that patients with a lower serum 25(OH)D level had a worse kidney outcome. More specifically, in pairwise comparison using the Log-rank test, the *P*-value was 0.001 (the lowest vs. middle tertile), <0.001 (the lowest vs. highest tertile), and 0.008 (the middle vs. highest tertile), respectively. The association between the 25(OH)D level and risks for composite kidney outcomes were further determined by Cox proportional hazards regression model (**Table 5**). In unadjusted models, compared with the highest tertile, the risk of kidney outcomes was higher in the lowest tertile [HR, 6.3 (3.2, 12.4), *P <* 0.001] and also relatively higher in the middle tertile [HR, 2.6 (1.2, 5.4), *P =* 0.01] (model 1 in **Table 5**). After adjustment for baseline eGFR, we also observed a significantly greater risk for CKD progression in the lowest tertile of the serum 25(OH)D level [HR, 5.2 (2.5, 10.7), *P <* 0.001] compared with the highest tertile of the serum 25(OH)D level (model 2 in **Table 5**). This increased risk remained in the lowest tierce, even after extensive adjustment for baseline age, gender, HbA1c, 24-h urinary protein, systolic blood pressure, use of RAASi, oral hypoglycemic agents, and insulin [HR, 3.2 (1.3, 7.8), *P =* 0.01, model 3 in **Table 5**, **Figure 4**].

**Table 5:** Effect of serum 25(OH)D on renal outcomes.

Variable	Serum 25(OH)D			*P*-value for trend
	T1 (≤17) (n = 60)	T2 (17–35) (n = 62)	T3 (>35) (n = 60)	
**Number of events, %**	39 (65)	20 (32)	11 (18)	**<0.001**
**Model 1 HR (95%CI)**	6.3 (3.2, 12.4)	2.6 (1.2, 5.4)	1 [reference]	**<0.001**
***P*-value**	**<0.001**	**0.01**		
**Model 2 HR (95%CI)**	5.2 (2.5, 10.7)	2.3 (1.1, 4.8)	1 [reference]	**<0.001**
***P*-value**	**<0.001**	**0.04**		
**Model 3 HR (95%CI)**	3.2 (1.3, 7.8)	1.7 (0.8, 3.9)	1 [reference]	**0.03**
***P*-value**	**0.01**	0.19		

Hazard ratios (HR) and 95% confidence intervals were derived from Cox proportional hazards regression models.

CKD, chronic kidney disease; eGFR, estimated glomerular filtration rate; RAAS, renin-angiotensin-aldosterone system; HbA1c, glycosylated hemoglobin; SBP, systolic blood pressure.

Model 1: unadjusted.

Model 2: adjusted for eGFR.

Model 3: Model 2 plus age, gender, HbA1c, 24-h urinary protein, SBP, use of RAAS inhibitor, oral hypoglycemic agents, and insulin.A two-tailed P < 0.05 was considered statistically significant and presented in bold.

### Effect of TWA of the Serum 25(OH)D Level on Kidney Outcomes

**Figure 5:**
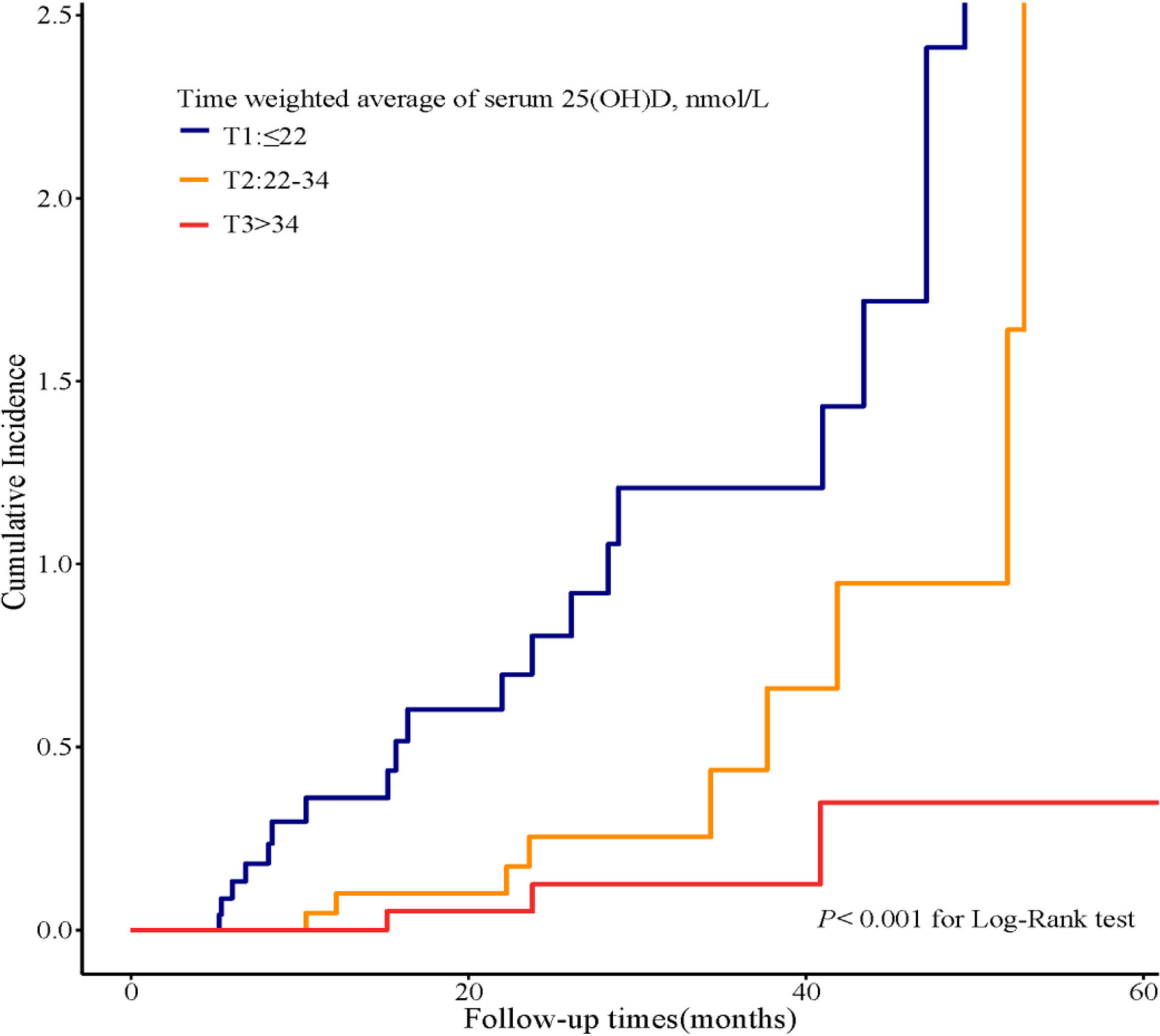
Cumulative incidence of CKD progression in strata of tertiles of TWA of the serum 25(OH)D levels. Kaplan–Meier curves compare different strata of tertiles of TWA of the serum 25(OH)D levels in enrolled patients. TWA, time weighted average.

Seventy-five patients received serum 25(OH)D measurements from two to nine times during follow-up. To substantiate our findings, we calculated the TWA of serum 25(OH)D in these 75 patients and further performed sensitivity analyses. We divided the patients into tertiles according to TWA of the serum 25(OH)D level (**Supplementary Tables 1, 2**). During a median follow-up time of 41 months (IQR, 24; 52 months), 31 outcome events occurred, including four events of D-Scr and 27 events of ESKD. Compared with the lowest tertile of TWA, Kaplan–Meier analyses demonstrated that the cumulative incidence of kidney outcomes was significantly lower in the highest tertile of TWA of the serum 25(OH)D level (*P <* 0.001) (**Figure 5**). In addition, we created a Cox proportional hazards regression model with tertiles of TWA of the serum 25(OH)D level in the same manner as above. Patients in the lowest tertile of TWA of the serum 25(OH)D level were associated with a higher risk for CKD progression [HR, 9.5 (2.8, 32.7), *P <* 0.001, model 1 in **Table 6**] compared with those in the highest tertile. This association remained significant after adjustment for baseline eGFR in model 2 [HR, 8.6 (2.5, 30.1), *P =* 0.001 for the lowest tertile, model 2 in **Table 6**]. After adjustment for baseline age, gender, HbA1c, 24-h urinary protein, systolic blood pressure, use of RAASi, oral hypoglycemic agents, and insulin, the risk for kidney outcomes increased with the reduction of 25(OH)D level (*P* for trend = 0.02, model 3 in **Table 6**).

**Table 6:** Effect of time weighted average of serum 25(OH)D on renal outcomes.

Variable	TWA of serum 25(OH)D			*P*-value for trend
	T1 (≤22) (n = 25)	T2 (22–34) (n = 25)	T3 (>34) (n = 25)	
**Number of events, %**	39 (65)	20 (32)	11 (18)	**<0.001**
**Model 1 HR (95%CI)**	9.5 (2.8, 32.7)	3.2 (0.9, 11.8)	1 [reference]	**<0.001**
***P*-value**	**<0.001**	0.08		
**Model 2 HR (95%CI)**	8.6 (2.5, 30.1)	2.5 (0.6, 9.7)	1 [reference]	**<0.001**
***P*-value**	**0.001**	0.18		
**Model 3 HR (95%CI)**	3.7 (0.8, 17.4)	1.0 (0.2, 5.2)	1 [reference]	**0.02**
***P*-value**	0.10	0.96		

Hazard ratios (HR) and 95% confidence intervals were derived from Cox proportional hazards regression models.

TWA, time weighted average; CKD, chronic kidney disease; eGFR, estimated glomerular filtration rate; RAAS, renin-angiotensin-aldosterone system; HbAc1, glycosylated hemoglobin; SBP, systolic blood pressure.

Model 1: unadjusted.

Model 2: adjusted for eGFR.

Model 3: Model 2 plus age, gender, HbA1c, 24-h urinary protein, SBP, use of RAAS inhibitor, oral hypoglycemic agents, and insulin.A two-tailed P < 0.05 was considered statistically significant and presented in bold.

## Discussion

The current study was specifically powered on the serum 25(OH)D level and T2DM with CKD. The principal finding of this study is that lower serum 25(OH)D level was significantly associated with an increased risk of CKD progression in patients with T2DM. This association was independent of other established important covariables, including baseline eGFR, age, HbA1c, 24-h urinary protein, blood pressure, use of RAASi, oral hypoglycemic agents, and insulin. In addition, these relationships remained robust with further sensitivity analysis of data with TWA of the serum 25(OH)D level, showing an independent association between lower TWA of the serum 25(OH)D level and an unfavorable kidney outcome in patients with T2DM with CKD.

Surveys conducted in Chinese cities among patients with T2DM reported the proportions of VD deficiency were about 62.7%–83.5% (27–29). In addition, VD deficiency may be a prominent element of CKD due to that reduced CYP27B1 activity in human renal PTECs inhibits the production of 1,25(OH)2D and impairs the function of reabsorption of 25(OH)D (6, 30). Here, we reported 87.4% [25(OH)D <50 nmol/L], 40.4% [25(OH)D <25 nmol/L], or 58.8% [25(OH)D <30 nmol/L] of patients with T2DM with CKD in Nanjing, which is located in eastern coastal China (latitude 31°–33°N), had VD deficiency according to those international guidelines. By dividing into pathological subgroups, up to 91.5% of patients with DKD were affected by VD deficiency and insufficiency, presenting decreased the serum 25(OH)D levels than those with NDKD. The findings suggested VD homeostasis might be related to the etiology and pathogenesis of DKD in patients with T2DM. The Third National Health and Nutrition Examination Survey (NHANES III) to assess people with diabetes demonstrated an independent association between VD deficiency and DKD (31). In addition, the insufficiency of VD is more serious when DKD is progressing (32).

Previous studies were mainly focused on T2DM population to explore the relationship of VD levels and the presence of DKD (12, 13, 28). Recent studies have indicated that hypovitaminosis D was associated with a higher risk of developing DKD in T2DM (15). The included population of our current study was patients with T2DM complicated with biopsy-proven CKD, which was allowed to assess the clinical and pathological features in strata of VD levels and better illustrate the relationship between the development of renal function through pre-dialysis stages with different levels of VD. More importantly, our data strongly suggest that in patients with T2DM complicated with CKD, lower serum 25(OH)D levels were associated with an increased risk of CKD progression. The findings are robust because we showed consistent results with baseline and time-updated patterns of 25(OH)D levels. Patients with both lower baseline and TWA of 25(OH)D levels were almost three times risk to CKD progression compared with those with higher 25(OH)D levels after adjustment for multiple risk factors. This is the first study in patients with T2DM with CKD highlighting TWA of 25(OH)D levels, representing a sensitivity analysis that supports our primary hypothesis regarding the association between lower 25(OH)D levels and adverse kidney-related outcomes. These results might suggest that the long-term maintenance of optimal VD concentrations early in life has been associated with reduced future risk of CKD development in T2DM.

In the present study, we observed that 25(OH)D levels were positively correlated with serum calcium, 24-h urinary calcium and phosphorus excretion, whereas negatively correlated with PTH level and vascular lesion score. The VD endocrine system is critical for human health. VD and its active metabolite are steroid hormones, contributing to regulating the metabolism of calcium and phosphate and playing a critical role in maintaining bone health (33). The best characterized features of CKD associated with VD deficiency are defects in mineral metabolism, including intestinal calcium absorption and renal phosphate excretion (34). Low serum 25(OH)D levels contribute to reduced 1,25(OH)2D levels by providing less substrate for conversion (35). Subsequently, lower 1,25(OH)2D levels reduce calcium absorption from the gastrointestinal tract, promoting PTH secretion, which is associated with abnormal bone remodeling and the propensity to vascular calcifications (30, 34). Therefore, in the present study, the 25(OH)D levels were positively associated with serum calcium and 24-h urinary calcium excretion, whereas negatively correlated with PTH level and vascular lesion score. In addition, VD, PTH, FGF 23, and klotho form a complex endocrine network to maintain phosphate homeostasis (34). Previous studies reported that active VD induces expression of the FGF23 and α-klotho genes to attenuate the pro-aging effects of hyperphosphatemia and maintain the plethora of anti-aging and pro-survival actions of renal and circulating klotho (34). Taken together, in T2D with CKD, low 25(OH)D levels may have a role in electrolyte imbalance and vascular lesions, which need further well-designed studies to elucidate the mutual relationship and their detailed molecular mechanisms.

Several limitations of our study should be considered. First, the current study is observational in nature. It precludes conclusions concerning causality and cannot exclude the possibility of residual confounding. We conducted an additional multivariable logistic regression analysis with TWA of 25(OH)D levels to further ameliorate the imbalance in potential confounders. However, some bias inherent to retrospective studies may play. Second, this study was conducted in a single center, and the sample size was limited. Third, although serum and 24-h urinary calcium and phosphorus levels, PTH, and renin-angiotensin blocker were analyzed, other potential confounding factors affecting 25(OH)D levels were not included, such as outdoor exercise, sun exposure, nutritional status, seasonal alternation, dietary habits, and bone metabolism markers. In addition, direct measurement of free 25(OH)D and DBP were not routinely performed in clinical practice.

In conclusion, our data suggested that patients with T2DM with a decreased 25(OH)D level had deteriorated renal function. Both lower baseline and TWA of the serum 25(OH)D levels were associated with increased risk of CKD progression in patients with T2DM after adjusting numerous potential confounders, which suggested that the long-term maintenance of optimal VD levels from early in life might be associated with reduced future risk of CKD development in T2DM.

## Data Availability Statement

The raw data supporting the conclusions of this article will be made available by the authors, without undue reservation.

## Ethics Statement

The studies involving human participants were reviewed and approved by the Ethics Committee of The First Affiliated Hospital of Nanjing Medical University. The patients/participants provided their written informed consent to participate in this study.

## Author Contributions

SD designed the research and contributed to the writing. FL analyzed the data and performed statistical analysis. BW, CZ, GN, LS, ZH, and HG reviewed the manuscript. CX and BZ conceived and coordinated the study and had responsibility for its final content. YY is the guarantor of this work who had complete access to all the data in the study and takes ultimate responsibility for the study design and integrity of data analysis. All authors contributed to the article and approved the submitted version.

## Conflict of Interest

The authors declare that the research was conducted in the absence of any commercial or financial relationships that could be construed as a potential conflict of interest.

## Publisher’s Note

All claims expressed in this article are solely those of the authors and do not necessarily represent those of their affiliated organizations, or those of the publisher, the editors and the reviewers. Any product that may be evaluated in this article, or claim that may be made by its manufacturer, is not guaranteed or endorsed by the publisher.
