# Supplementary material for: Association of Serum 25 (OH) Vitamin D With Chronic Kidney Disease Progression in Type 2 Diabetes
Source: Front Endocrinol (Lausanne). 2022 Jun 30;13:929598. doi: 10.3389/fendo.2022.929598 (PMC9279917; doi:10.3389/fendo.2022.929598)
Supplement: Supplementary file 1 [file Table_1.docx]

Supplemental Table 1. Clinical characteristics and laboratory findings according to tertiles of TWA of serum 25(OH)D level.

| **Parameter** | **Total (n=75)** | **Tertiles of TWA of serum 25(OH)D level (nmol/L)** | | | ***P* value** |
| --- | --- | --- | --- | --- | --- |
|  |  | **T1 (≤22) (n=25)** | **T2 (22-34) (n=25)** | **T3 (>34) (n=25)** |  |
| Age (years) | 52±12 | 48±10 | 54±12 | 52±12 | 0.16 |
| Gender (male/female) | 55/20 | 19/6 | 15/10 | 21/4 | 0.15 |
| **Comorbid disease** |  |  |  |  |  |
| CKD stage (1/2/3a/3b/4) | 43/60/23/27/29 | 7/17/11/12/13 | 15/20/7/10/10 | 21/23/5/5/6 | **<0.001** |
| Duration of diabetes (years) | 8.0 (3.0,10.5) | 9.0 (3.0,15.0) | 8.0 (6.0,11.0) | 7.0 (2.0,10.0) | 0.37 |
| Diabetic retinopathy (%) | 17 (23) | 7 (28) | 5 (20) | 5 (20) | 0.83 |
| Diabetic neuropathy (%) | 6 (8.0) | 2 (8.0) | 3 (12) | 1 (4.0) | 0.87 |
| Hypertension (%) | 54 (72) | 17 (68) | 18 (72) | 19 (76) | 0.82 |
| **Clinical parameter** |  |  |  |  |  |
| Body mass index (kg/m^2^) | 25±3.3 | 26±2.7 | 24±2.5 | 26±4.1 | **0.02** |
| SBP (mmHg) | 146±22 | 149±25 | 147±23 | 141±16 | **0.03** |
| DBP (mmHg) | 85±14 | 87±18 | 84±14 | 83±9.0 | 0.98 |
| MAP (mmHg) | 105±15 | 107±19 | 105±15 | 103±8.8 | 0.64 |
| **Laboratory parameter** |  |  |  |  |  |
| Urinary protein excretion (g/d) | 3.0 (1.5,7.5) | 3.5 (1.6,9.4) | 6.5 (2.7,10) | 0.63 (0.20,2.3) | **<0.001** |
| eGFR (ml/min/1.73 m²) | 67±30 | 60±23 | 60±34 | 80±28 | **0.001** |
| BUN (mmol/L) | 8.5 (6.4,11) | 8.4 (6.3,9.6) | 12 (10,14) | 8.1 (5.7,15) | **0.01** |
| Scr (μmol/L) | 102 (77,159) | 104 (102,156) | 138 (83,219) | 96 (77,157) | 0.05 |
| Uric acid (mmol/L) | 371±94 | 374±81 | 361±103 | 378±101 | 0.81 |
| Serum albumin (g/L) | 32±7.9 | 27±7.4 | 31±6.4 | 37±6.7 | **<0.001** |
| ALP(U/L) | 79 (59,106) | 90 (71,107) | 59 (56,72) | 71 (59,95) | 0.08 |
| Serum 25(OH)D (nmol/L) | 24 (14,38) | 15 (8.2,19) | 29 (18,35) | 35 (32,83) | **<0.001** |
| Serum calcium (mmol/L) | 2.10±0.19 | 2.04±0.17 | 2.07±0.20 | 2.19±0.15 | **0.02** |
| Serum phosphorus (mmol/L) | 1.24±0.23 | 1.21±0.20 | 1.28±0.23 | 1.22±0.27 | 0.53 |
| 24h urinary calcium  (mmol/d) | 2.08 (0.95,3.95) | 1.62 (1.19,3.18) | 2.28 (0.97,3.42) | 1.67 (0.88,3.57) | 0.56 |
| 24h urinary phosphorus  (mmol/d) | 15.8 (12.3,21.9) | 13.8 (11.1,19.7) | 17.9 (15.8,20.2) | 17.9 (11.7,18.1) | 0.35 |
| time weighted average of serum 25(OH)D | 28 (17,42) | 14 (11,18) | 29 (28,31) | 50 (45,63) | **<0.001** |
| FBG (mmol/L) | 6.4 (4.5,8.3) | 7.9 (6.0,12.0) | 6.0 (3.3,8.7) | 4.8 (4.3,9.5) | 0.20 |
| HbA1c (%) | 7.0 (6.4,8.1) | 8.6 (7.2,10.4) | 7.2 (7.0,8.2) | 6.3 (6.0,7.0) | 0.25 |
| TG (mmol/L) | 1.7 (1.2, 2.6) | 1.7 (1.1,3.7) | 1.6 (1.1,3.0) | 1.6 (1.1,2.0) | 0.23 |
| TC (mmol/L) | 5.4±1.8 | 6.3±1.8 | 5.5±1.8 | 4.6±1.3 | **0.002** |
| LDL-C (mmol/L) | 3.4±1.2 | 3.9±1.1 | 3.4±1.3 | 3.0±1.0 | **0.01** |
| HDL-C (mmol/L) | 1.05 (0.87,1.42) | 1.11 (0.93,1.50) | 1.15 (0.86,1.39) | 0.94 (0.72,1.02) | 0.15 |
| Hemoglobin(g/L) | 115±21 | 111±18 | 108±16 | 125±24 | **0.007** |
| PTH (pg/mL) | 42 (24,67) | 31 (24,38) | 23 (17,43) | 46 (28,67) | 0.73 |
| Serum IgA (g/L) | 2.3 (1.7,3.0) | 3.1 (2.5,3.9) | 2.1 (1.7,2.5) | 1.9 (1.6,2.8) | 0.20 |
| Serum IgG (g/L) | 10±3.9 | 9.0±3.5 | 10±4.6 | 11±3.5 | 0.21 |
| Serum C3 (g/L) | 1.09±0.25 | 1.15±0.23 | 1.07±0.25 | 1.04±0.26 | 0.26 |
| Serum C4 (g/L) | 0.31±0.10 | 0.35±0.08 | 0.29±0.10 | 0.28±0.10 | **0.03** |
| Serum NAGL (ng/mL) | 194 (143,355) | 178 (76,226) | 182 (152,196) | 236 (154,499) | 0.35 |
| Urinary NAGL (ng/mL) | 54 (25,93) | 25 (25,67) | 54 (25,96) | 25 (25,58) | **0.02** |
| 24-hour uNAG (U/L) | 14 (10,23) | 21 (21,32) | 10 (8.1,15) | 10 (7.8,12) | **<0.001** |
| RBP (mg/L) | 61±23 | 60±19 | 68±27 | 56±21 | 0.20 |
| **Medications** |  |  |  |  |  |
| RAAS inhibitor (%) | 62 (83) | 21 (84) | 19 (76) | 22 (88) | 0.65 |
| Oral hypoglycemic agents (%) | 38 (50) | 12 (48) | 11 (44) | 15 (60) | 0.59 |
| Insulin (%) | 54 (72) | 20 (80) | 19 (76) | 15 (60) | 0.36 |
| β-blocker (%) | 21 (28) | 8 (32) | 6 (24) | 7 (28) | 0.95 |
| Diuretic (%) | 6 (8.0) | 2 (8.0) | 2 (8.0) | 2 (8.0) | 1.0 |
| CCB (%) | 48 (64) | 15 (60) | 15 (60) | 18 (72) | 0.72 |
| Statins (%) | 31 (41) | 12 (48) | 12 (48) | 7 (28) | 0.30 |
| **Progression** |  |  |  |  |  |
| Composite renal outcome (%) | 31 (41) | 19 (76) | 9 (36) | 3 (12) | **<0.001** |
| D-Scr (%) | 4 (5.3) | 3 (12) | 1 (4.0) | 0 (0) | 0.11 |
| ESKD (%) | 27 (36) | 16 (64) | 8 (32) | 3 (12) | **0.001** |

TWA time weighted average, CKD chronic kidney disease; BMI body mass index; SBP systolic blood pressure; DBP diastolic blood pressure; MAP mean arterial pressure MAP = (systolic blood pressure+2*diastolic blood pressure)/3; eGFR estimated glomerular filtration rate; BUN blood urea nitrogen; Scr serum creatinine; ALP [alkaline](javascript:;) [phosphatase](javascript:;); 25(OH)D 25-hydroxyvitamin D; FBG fasting blood glucose; HbA1c glycosylated Hemoglobin; TG triglyceride; TC total cholesterol; LDL-C low-density lipoprotein cholesterol; HDL-C high-density lipoprotein cholesterol; PTH parathyroid hormone; IgA immunoglobulin A; IgG immunoglobulin G; C3 complement 3; C4 complement 4; NAGL neutrophil gelatinase-associated lipocalin; uNAG urinary N-acetyl-β-D glucosaminidase; RBP retinol binding protein; RAAS renin-angiotensin-aldosterone system; CCB calcium-channel blocker. D-Scr doubling of serum creatinine level; ESKD end stage kidney disease;

Data were presented as the mean±standard, the median with interquartile range or counts and percentages. A two-

tailed *P*<0.05 was considered statistically significant.

Supplemental Table 2. Pathological features according to tertiles of TWA of serum 25(OH)D level.

| **Pathological feature** | **Total (n=75)** | **Tertiles of TWA of serum 25(OH)D level(nmol/L)** | | | ***P* value** |
| --- | --- | --- | --- | --- | --- |
|  |  | **T1 (≤22) (n=25)** | **T2 (22-34) (n=25)** | **T3 (>34) (n=25)** |  |
| **DKD/NDKD (cases)** | 53/22 | 22/3 | 18/7 | 13/12 | **0.02** |
| **Glomerular class of DKD(I/IIa/IIb/III/IV)** | 0/6/8/32/7 | 0/1/1/16/2 | 0/1/3/9/5 | 0/4/2/7/0 | **0.04** |
| **Pathological**  **classiﬁcation of NDKD** |  |  |  |  |  |
| IgAN/MCD/FSGS/MN/LN/CGN (cases) | 7/1/4/7/1/2 | 1/0/0/2/0/0 | 1/0/2/4/0/2 | 5/1/2/1/1/2 | 0.11 |
| **IFTA Score (0/1/2/3)** | 7/28/17/22 | 1/7/6/10 | 3/6/7/9 | 3/15/4/3 | **<0.001** |
| DKD subtype | 2/16/15/20 | 1/6/6/9 | 1/2/6/9 | 0/8/3/2 | **<0.001** |
| NDKD subtype | 5/12/2/2 | 0/1/0/1 | 2/4/1/0 | 3/7/1/1 | **0.02** |
| **Interstitial inflammation (0/1/2/3)** | 11/45/18/0 | 3/16/5/0 | 4/14/7/0 | 4/15/6/0 | **0.008** |
| DKD subtype | 7/33/13/0 | 3/14/5/0 | 2/10/6/0 | 2/9/2/0 | **0.006** |
| NDKD subtype | 4/12/5/0 | 0/2/0/0 | 2/4/1/0 | 2/6/4/0 | **0.04** |
| **Vascular lesion Score (0/1/2)** | 19/23/32 | 5/6/13 | 4/9/12 | 10/8/7 | **<0.001** |
| DKD subtype | 6/15/32 | 3/6/13 | 0/6/12 | 3/3/7 | **0.002** |
| NDKD subtype | 13/8/0 | 2/0/0 | 4/3/0 | 7/5/0 | 0.14 |
| **Global sclerosis, %** | 25 (7.3,42) | 23 (13,38) | 33 (7.7,50) | 14 (2.3,27) | 0.12 |
| DKD subtype | 25 (9.6,45) | 23 (13,38) | 38 (11,65) | 14 (6.9.3) | 0.21 |
| NDKD subtype | 21 (0.0,33) | 32 (31,43) | 15 (6.7,33) | 20 (0.0,33) | 0.44 |
| **Glomerular IgG deposition (0/1/2/3)** | 47/17/3/6 | 14/6/2/2 | 15/6/1/2 | 18/5/0/2 | **0.001** |
| DKD subtype | 36/14/2/1 | 13/6/2/1 | 13/5/0/0 | 10/3/0/0 | **0.005** |
| NDKD subtype | 11/3/1/5 | 1/0/0/1 | 2/1/1/2 | 8/2/0/2 | 0.06 |
| **Glomerular IgM deposition, (0/1/2/3)** | 16/10/17/30 | 3/2/8/11 | 4/6/5/9 | 9/2/4/10 | **<0.001** |
| DKD subtype | 11/7/13/22 | 3/2/7/10 | 4/3/5/6 | 4/2/1/6 | **<0.001** |
| NDKD subtype | 5/3/4/8 | 0/0/1/1 | 0/3/0/3 | 5/0/3/4 | 0.47 |
| **Glomerular IgA deposition, (0/1/2/3)** | 41/10/8/14 | 13/5/3/3 | 13/4/3/4 | 15/1/2/7 | **<0.001** |
| DKD subtype | 32/9/6/6 | 12/5/3/2 | 11/3/2/2 | 9/1/1/2 | **0.001** |
| NDKD subtype | 9/1/2/8 | 1/0/0/1 | 2/1/1/2 | 6/0/1/5 | 0.07 |
| **Glomerular C3 deposition (0/1/2/3)** | 36/9/9/19 | 12/2/4/6 | 10/4/1/9 | 14/3/4/4 | **<0.001** |
| DKD subtype | 28/5/8/12 | 12/2/4/4 | 8/2/1/7 | 8/1/3/1 | **<0.001** |
| NDKD subtype | 8/4/1/7 | 0/0/0/2 | 2/2/0/2 | 6/2/1/3 | **0.02** |
| **Glomerular** **C4 deposition (0/1/2/3)** | 52/11/4/6 | 14/6/2/2 | 18/2/0/4 | 20/3/2/0 | **<0.001** |
| DKD subtype | 37/9/2/5 | 13/6/2/1 | 13/1/0/4 | 11/2/0/0 | **<0.001** |
| NDKD subtype | 15/2/2/1/ | 1/0/0/1 | 5/1/0/0 | 9/1/2/0 | **0.02** |
| **Glomerular C1q deposition (0/1/2/3)** | 46/14/7/6 | 12/7/2/3 | 16/2/4/2 | 18/5/1/1 | **<0.001** |
| DKD subtype | 36/8/5/4 | 12/6/2/2 | 13/0/3/2 | 11/2/0/0 | **<0.001** |
| NDKD subtype | 10/6/2/2 | 0/1/0/1 | 3/2/1/0 | 7/3/1/1 | **0.01** |

TWA time weighted average, DKD diabetic kidney disease; NDKD non-diabetic kidney disease; IgAN IgA nephropathy; MCD minimal change disease; FSGS Focal segmental glomerulosclerosis; MN [membranous](javascript:;) [nephropathy](javascript:;); LN [lupus](javascript:;) [nephritis](javascript:;); CGN crescentic glomerulonephritis; IFTA interstitial inflammation; A two-tailed *P*<0.05 was considered statistically significant.
